# Herb-partitioned moxibustion alleviates colonic inflammation in Crohn’s disease rats by inhibiting hyperactivation of the NLRP3 inflammasome via regulation of the P2X7R-Pannexin-1 signaling pathway

**DOI:** 10.1371/journal.pone.0252334

**Published:** 2021-05-27

**Authors:** Ji Zhang, Xue-jun Wang, Li-jie Wu, Ling Yang, Yan-ting Yang, Dan Zhang, Jue Hong, Xi-ying Li, Xiao-qing Dong, Xiao-cong Guo, Rong Han, Xiaopeng Ma

**Affiliations:** 1 Yueyang Clinical Medicine School, Shanghai University of Traditional Chinese Medicine, Shanghai, China; 2 Acupuncture and Moxibustion Department, Zhejiang Provincial Hospital of Traditional Chinese Medicine, Hangzhou, Zhejiang, China; 3 Key Laboratory of Acupuncture-Moxibustion and Immunology, Shanghai Research Institute of Acupuncture and Meridian, Shanghai University of Traditional Chinese Medicine, Shanghai, China; Universite Paris-Sud, FRANCE

## Abstract

Crohn’s disease is a chronic inflammatory bowel disease and the NLRP3 inflammasome plays an important role in Crohn’s disease. Previous studies have shown that Herb-partitioned moxibustion treating (at Qihai (CV 6) and Tianshu (ST 25)) prevented the excessive activation of the NLRP3 inflammasome and repaired damaged colonic mucosa in Crohn’s disease. However, the mechanism by which Herb-partitioned moxibustion (at CV 6 and ST 25) regulates NLRP3 remains unclear. In this study, we treated Crohn’s disease rats with herb-partitioned moxibustion (at CV 6 and ST 25) to investigate the mechanism by which Herb-partitioned moxibustion regulates the colonic NLRP3 inflammasome by observing colon length, the colon macroscopic damage indexes, and the expression of ATP, P2X7R, Pannexin-1, NF-κBp65, NLRP3, ASC, caspase-1, IL-1β and IL-18 in the colon in Crohn’s disease. Here, this study shows that herb-partitioned moxibustion (at CV 6 and ST 25) can reduce colon macroscopic damage indexes and colon histopathological scores, alleviate colon shortening and block the abnormal activation of the NLRP3 inflammasome by inhibiting the ATP content and the expression of P2X7R, Pannexin-1 and NF-κBp65, thereby reducing the release of the downstream inflammatory cytokine IL-1β and ultimately suppressing colonic inflammation in Crohn’s disease rats. This study for the first time identifies the mechanism by which herb-partitioned moxibustion (at CV 6 and ST 25) may inhibit the abnormal activation of the NLRP3 inflammasome by inhibiting the P2X7R-Pannexin-1 signaling pathway in Crohn’s disease rats.

## Introduction

Crohn’s disease (CD) is a chronic inflammatory bowel disease that first manifests as typical clinical symptoms of diarrhea, abdominal pain, perianal fistulas, abdominal mass, and bowel obstruction [1]. Extraintestinal manifestations of CD are frequent and include peripheral arthritis, erythema nodosum, primary sclerosing cholangitis, episcleritis and so on [2]. At present, there is no cure for CD. Some patients choose surgery [3], but surgical intervention for CD requires further study because it is associated with a high recurrence rate [4, 5]. Medications used to maintain remission include 5-aminosalicylic acid products, corticosteroids and immunomodulators. However, all of these medications have adverse effects [6, 3]. As research has progressed, the use of biologics (anti-TNF-a) and stem cell therapies has substantially improved CD treatment [7–9], but anti-TNF therapy may increase the risk for serious and opportunistic infections in patients, and stem cell therapies are expensive and limited by the challenges of stem cell transport [10, 11]. Clinical studies have demonstrated that herb-partitioned moxibustion (HPM) is effective in treating CD [12, 13]. HPM exerts its therapeutic effects by regulating intestinal epithelial barrier function [14–16], autophagy and immune function in colon tissues [17] and it is known for to affect multiple targets, have multiple effects and exhibit high safety.

The pathogenesis of CD remains unclear, although genetic susceptibility, environmental factors, altered gut microbiota and abnormal mucosal immune reactions have all been found to contribute to the pathogenesis of CD [1, 6, 18]. Over the past two decades, studies have reported that susceptibility to CD is associated with abnormalities in NOD2 and the NLRP3 inflammasome [19]. The NLRP3 inflammasome consists of a sensor, i.e., NLRP3; an adaptor, i.e., apoptosis-associated speck-like protein (ASC); and an effector protein, i.e., caspase-1. Activation of the NLRP3 inflammasome induces the maturation and production of the proinflammatory cytokines interleukin 1β (IL-1β) and IL-18, which further induce inflammation in inflammatory bowel diseases [20].

**Fig 1:**
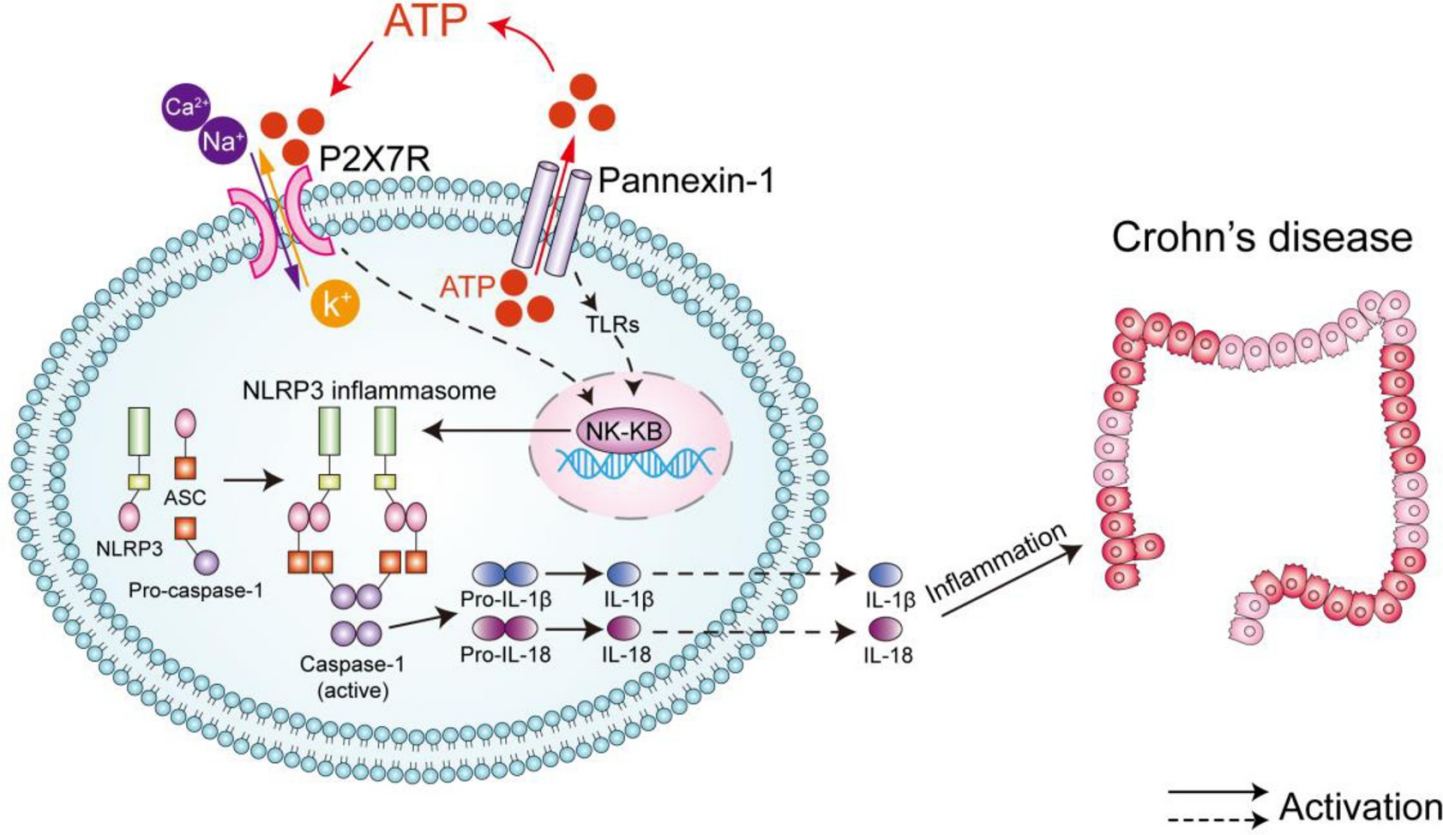
Schematic diagram illustrating the mechanism underlying the activation of the NLRP3 inflammasome by the ATP/P2X7R-Pannexin-1 signaling pathway in CD. Note: ATP/P2X7R-Pannexin-1 signaling pathway induces the activation of NF-κB, which causes the transcription of NLRP3 mRNA. When NLRP3 is activated, ASC recruits pro-caspase-1 to assemble into a large cytosolic complex via the CARD domain, triggering the activation of caspase-1. Subsequently, active caspase-1 cleaves pro-IL-1β and pro-IL-18 and then releases biologically active mature IL-1β and IL-18, which induce inflammatory and autoimmune conditions including CD.

The NLRP3 inflammasome can be activated by P2X7 receptor (P2X7R) and then induce the secretion of IL-1β and IL-18 [21]. At inflammatory sites, where extensive immune cell activation and tissue damage cause the release of large amounts of extracellular ATP, P2X7R can be activated by high concentrations of extracellular ATP [22]. The combination of extracellular ATP and P2X7R allows the passage of cations, including Ca^2+^, Na^+^, and K^+^, across the plasma membrane [23, 24], and then recruits Pannexin-1 to mediate the opening of hemichannels and promote the release of intracellular ATP [25]. Subsequently, P2X7R and Pannexin-1 channels induce the activation of nuclear factor-κB (NF-κB), which may cause the transcription of NLRP3 mRNA [26–28]. When NLRP3 is activated, ASC recruits pro-caspase-1 to assemble into a large cytosolic complex via the CARD domain, triggering the activation of caspase-1. Subsequently, active caspase-1 cleaves pro-IL-1β and pro-IL-18 and then releases biologically active mature IL-1β and IL-18, which induce chronic intestinal inflammation [20] (Fig 1).

Previous study showed that herb-partitioned moxibustion (HPM) (at CV 6 and ST 25) improved increased cell apoptosis in the intestinal epithelial barrier, alleviated intestinal epithelial tight junction integrity, prevented the excessive activation of the NLRP3 inflammasome and reduce the expression of IL-1β in the colons of CD rats to promote the repair of colon damage [14–16, 29]. However, whether HPM (at CV 6 and ST 25) can regulate the P2X7R-Pannexin-1 signaling pathway to inhibit the abnormal activation of the NLRP3 inflammasome in CD is unclear. Thus, we treated TNBS-induced experimental colitis with HPM to investigate the molecular mechanism by which HPM regulates the colonic NLRP3 inflammasome by observing the ATP content and the expression of P2X7R, Pannexin-1 and NF-κBp65 (Fig 1).

## Materials and methods

### Animals

Sprague-Dawley rats (150±20 g) were obtained from Shanghai SLAC Laboratory Animal Co., Ltd. (Shanghai, China; License no.: SCXK (Shanghai) 2017–0005). All rats were raised at Fudan University (18–26°C, 40%-70% humidity and a 12-h light/dark cycle). The animal protocols were approved by the Ethics Committee of Fudan University (no. 201811002Z).

### Reagents and equipment

The following reagents were used: 2, 4, 6-Trinitrobenzene sulfonic acid (TNBS, P2297), Brilliant Blue G (BBG, B0770) and pentobarbital sodium (Sigma, St Louis, MO, USA); aconite (Lot number: 20080403, Huaji Pharmaceutical Co. Ltd., Shanghai, China). aconite details: light taste, brown powder, low odor. absolute ethanol, methanol, xylene, isopropanol and chloroform (Sinopharm Chemical Reagent Co., Ltd., Shanghai, China); the ATP Assay Kit, hematoxylin and eosin, Interleukin -1β Assay Kit and Interleukin -18 Assay Kit (Nanjing Jiancheng Technology Co., Ltd., Nanjing, China); RIPA lysis buffer, HRP-labeled goat anti-rabbit IgG (H+L) and HRP-labeled goat Anti-mouse IgG (H+L), (Beyotime, Shanghai, China); a DakoREAL^TM^ EnVision^TM^ Rabbit/Mouse secondary antibody detection kit (k5007) (Dako, Glostrup, Denmark); a mouse anti-P2X7 monoclonal antibody (sc-514962), mouse anti-caspase-1 p10 monoclonal antibody (sc-56036) and mouse anti-ASC monoclonal antibody (sc-514414) (Santa Cruz, USA); a rabbit anti-Pannexin-1 polyclonal antibody (ab139715), rabbit anti-p-NF-κBp65 polyclonal antibody (ab86299), rabbit anti-NLRP3 polyclonal antibody (ab214185), rabbit anti-IL-1β polyclonal antibody (ab205924) and rabbit anti-IL-18 polyclonal antibody (ab191860) (Abcam, Cambridge, MA, USA); a rabbit anti-NF-κBp65 monoclonal antibody (8242S) and rabbit anti-GAPDH monoclonal antibody (2118S) (Cell Signaling Technology, Danvers, MA, USA); the SYBR Green PCR kit (#K0223) (Thermo Fisher Scientific, USA); and the Revert Aid First Strand cDNA Synthesis Kit (#K1622) (Thermo Fermentas, USA). The following equipment were used: an automatic hydroextractor, microtome, drying machine, freezing microtome and pathological analysis system (Leica, Wetzlar, Germany); a real-time PCR machine (ABI, USA); a light microscope and imaging system (Olympus, Tokyo, Japan); a gel imaging system (Bio-RAD, ChemiDocTMXPS^+^, USA); and a microplate reader (Bio Tek, SynergyTMH4, USA).

### Experimental grouping and establishment of a CD rat model

Ethical issues should be also considered while determining the sample size in animal studies. Russell and Burch (London: Methuen & Co. Ltd; 1959) in the Principles of Humane Experimental Technique (1959) proposed that the 3Rs are similar to ethical considerations applied to any animal experiment by researchers and other institutes conducting these studies [30]. In the case of animal ethics, it is proper to have 3–8 animals per group [31–34]. Therefore, in this study, 45 rats were randomly divided into five groups by SPSS 25.0 software, with 9 rats in each group: the normal group (NG), model group (MG), herb-partitioned moxibustion group (HPMG), brilliant blue G group (BBG-G) and sham moxibustion group (SMG). CD was induced in rats from all groups except those in the NG by administration of an enema containing a mixture of TNBS and ethanol as previously described [35]. Briefly, before model establishment, all rats were fasted and given only water for 24 h, weighed and anaesthetized using an intraperitoneal injection of 1% pentobarbital sodium (45 mg/kg). The TNBS mixture was instilled into the colon with a rubber cannula (3 mL/kg). The procedure was repeated every 7 days for 4 weeks. After the modeling process was finished, one animal from each group was randomly selected for H&E staining of colon tissue to determine whether the CD model was successfully established. Follow-up interventions were carried out only after it was confirmed that the model was successfully established.

### Treatment

**Fig 2:**
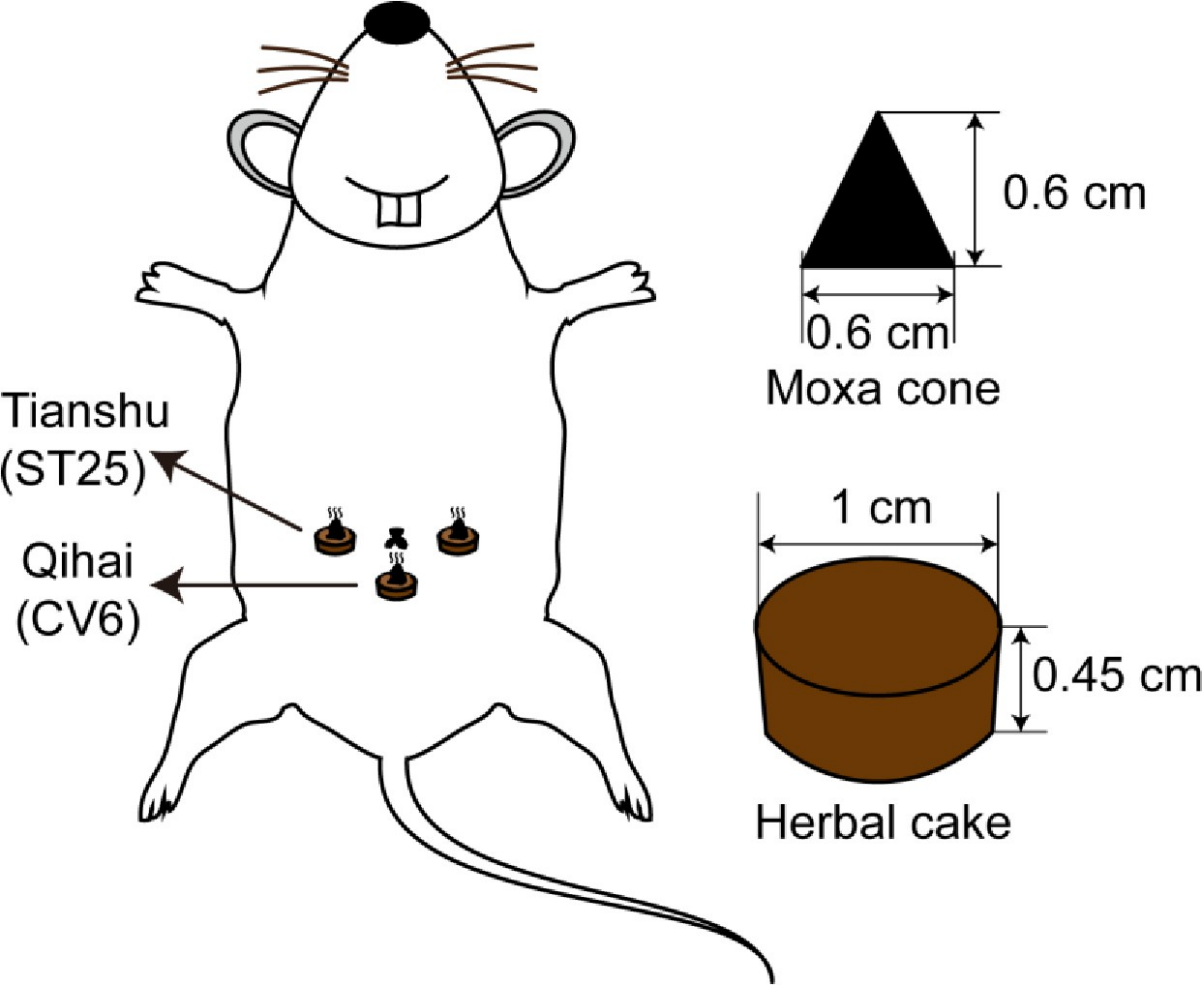
Illustration of herb-partitioned moxibustion. **Note:** HPM treatment was administered by placing a moxa cone on the top of an herbal cake at the Tianshu (ST25, bilateral) and Qihai (CV6) points and igniting it.

In the HPMG, treatment was administered by placing a moxa cone on the top of an herbal cake at the Tianshu (ST25, bilateral) and Qihai (CV6) points and igniting it (Fig 2). The treatment was administered for 10 min once daily for 7 days. The herbal cake was a thick paste made of Chinese medicine powder (aconite: aconitine (0. 0021%-0. 0670%), neoaconitine (0. 092%-0. 4%), hypoaconitine (0. 002%-0. 055 0%)) [36]. At room temperature, aconite powder (1.5g) mixed with yellow rice wine (1.5g) shaped using a mold (1 cm in diameter and 0.45 cm thick) (ratio: 1:1) and fixed it for 10 minutes. 0.0014415–0.00783g of targeted compound were included in final product. Other compounds (secokaraconitine, brachyaconitines A, C, talatisamine, songrine, bullatine A, 7-carbony sitosterone, lupeol, β-sitosterol and daucosterol) were included in the final product [37]. Research shows that compared with moxibustion, the thermal action of herbal cake moxibustion was modest and its radiation peak matched that at the acupuncture point. Thus, herbal cake moxibustion may act by producing modest thermal action and a sympathetic vibration at the skin surface [38]. The moxa cone was made by placing approximately 90 mg moxa wool in a mold (0.6 cm in diameter and 0.6 cm high). We used brilliant blue G (BBG) as a positive control drug for intervention and the rats in the BBG-G were intraperitoneally injected with BBG (50 mg/kg) once a day for 7 days. The rats in the SMG were subjected to the same treatment method as those in the HPMG without ignition of the moxa cone. The rats in the NG and MG did not receive any treatment. During the establishment of the model and the treatment period, the activity, mental status, eating and drinking of the rats in each group were observed twice a week, and the weight of the rats and the DAI scores were recorded [39].

### Sample collection and processing

After the intervention, all rats were anesthetized by intraperitoneal injection of 1% pentobarbital sodium (45 mg/kg). the injection was performed in the right caudal quadrant of the abdomen and approximately 5 mm to the right of midline. The needle was directed cranially at a 45° angle to the body wall. All rats were immediately euthanized by abdominal aorta exsanguination just after loss of consciousness. Until the rat loses the righting reflex and cessation of heartbeat, the next experiment can be carried out. Afterwards, the abdominal cavity was opened, the entire colon from the pubic symphysis to the distal cecum was collected, and the length of the colon was recorded [40]. The colon was then opened longitudinally and weighed after the feces were removed. Gross injury to the colon was observed, and colon macroscopic damage indexes (CMDIs) were determined [41]. The scores are shown in S1 Table. Subsequently, 6–8 cm of the distal colon located 2 cm from the anus was collected. Each colon was divided into five parts: two parts were stored in a -80°C freezer, one part was used as fresh tissue for the detection of ATP content, and the other two parts were fixed in 4% paraformaldehyde.

### Hematoxylin and eosin (H&E) staining

H&E was performed to observe histopathological injury in the colon. The colon tissues were fixed in formaldehyde, dehydrated, embedded in paraffin, sectioned at 4–5 μm, and stained with H&E. Histopathological injury in the colon tissues was observed under an optical microscope, and the pathological injury index was determined [42]. The detailed scoring criteria are shown in S2 Table.

### ATP content

ATP content in each group was measured using the ATP Assay Kit. In brief, colon tissues were homogenized by mechanical disruption in lysis buffer. The homogenates were centrifuged for 5 min at 12000 × g at 4°C, and the supernatants were collected for subsequent detection. Standard solutions of ATP and protein from the test samples were prepared to analyze colonic ATP content.

### Immunohistochemistry (IHC)

Immunohistochemistry was performed to evaluate the expression of NF-κBp65 in the colon according to the manufacturer’s specifications. Briefly, paraffin-embedded tissues were sliced into 5 μm-thick sections and heated at 60°C. Then, the sections were deparaffinized in xylene and gradient alcohol solutions (100%, 90%, 80%, and 70%), placed in citric acid buffer, and heated for antigen retrieval. After incubation in hydrogen peroxide solution for 25 min to reduce peroxidase activity, the sections were blocked with BSA for 25 min and stained with an anti-NK-kBp65 antibody (1:800) in a refrigerator overnight at 4°C. The next day, the sections were washed with PBS and reacted with 50 μL A solution (Dako Kit) for 30 min, and color development was performed with 1000 μL DAB solution. Finally, the sections were counterstained with hematoxylin, sealed and observed with a light microscope. Image-Pro-Plus was used for image analysis. From each slide, three visual fields were randomly selected to determine the mean integral optical density (IOD) for statistical analysis.

### Immunofluorescence (IF)

Sections were subjected to immunofluorescence to analyze Pannexin-1 expression. Prepared frozen tissue sections were rinsed with PBS to remove OCT embedding agent. A working solution of goat serum was used to block the sections for 1 h. After being rinsed with PBS, the sections were incubated with rabbit anti-Pannexin-1 primary antibody (1:250) overnight at 4°C. The next day, the sections were incubated with a secondary antibody at room temperature for 1 h, and then DAPI staining solution was applied to the sections for 10 min to stain the cell nuclei. The sections were observed under a fluorescence microscope, and images were obtained. The IOD intensity was quantified by Image-Pro-Plus.

### Western Blotting (WB)

Total protein was extracted from colon tissues, and the protein concentrations were measured with a BCA kit. Protein lysates were separated on an SDS-PAGE gel and transferred onto a PVDF membrane. Subsequently, the PVDF membrane was blocked for 2 h with 5% BSA at room temperature and incubated with primary antibodies overnight at 4°C. The next day, the membrane was washed four times in PBST and then incubated with a horseradish peroxidase (HRP)-conjugated secondary antibody (1:1000) for 1 h at room temperature. After the membrane was washed with PBST four times, the proteins were detected via the ECL chemiluminescence method. ImageJ software was used to analyze the gray values of the proteins.

The following primary antibodies were used: NLRP3 (1:300), ASC (1:500), caspase-1 (1:500), P2X7R (1:500), p-NF-κBp65 (1:2000), IL-1β (1:1000), IL-18 (1:1000), and GAPDH (1:1000).

### Real-time quantitative PCR analysis (RT-qPCR)

Colon tissues were treated with TRIzol reagent to extract total RNA following the manufacturer’s protocol (Invitrogen). Agarose gel electrophoresis was performed to measure RNA integrity, and RNA purity was assessed using a spectrophotometer. Subsequently, the RNA was used for reverse transcription with a DNA reverse transcription kit. The resultant cDNA was amplified with SYBR green PCR master mix. The PCR program was as follows: 95°C for 10 min followed by 40 cycles of 95°C for 15 s and 60°C for 45 s. The data were collected and analyzed with ABI Prism 7300 SDS software, and all data were analyzed by the 2^-△CT^ method (ΔCT = CT value of the target gene -CT value of the internal reference (GAPDH)) to calculate relative mRNA expression. The primer sequences are listed in Table 1.

**Table 1:** Primer sequences.

Gene	Primer F	Primer R	Size
P2X7R	5’ 5’ AGTTAGTACACGGCATCTTCG 3’	5’ 5’ GTGGGTCCATCCATCCTT 3’	183 bps
Pannexin-1	5’ GCCATACTCCTGTACCTGC 3’	5’ CAAGCTCCTCCATGATAAAC 3’	91 bps
NF-κBp65	5’ CCAAAGACCCACCTCACC 3’	5’ TGGCTAATGGCTTGCTCC 3’	163 bps
NLRP3	5’ ACCTCAACAGACGCTACAC 3’	5’ GTCCCACATCTTAGTCCTG 3’	119 bps
ASC	5’ TGCTGGATGCTCTGTATGG 3’	5’ CAAGTAGGGCTGTGTTTGC 3’	114 bps
caspase-1	5’ GACAAGCCCAAGGTTATC 3’	5’ GGCCTTCTTAATGCCATC 3’	105 bps
GAPDH	5’ GGAGTCTACTGGCGTCTTCAC 3’	5’ ATGAGCCCTTCCACGATGC 3’	237bps

### Enzyme-linked immunosorbent assay (ELISA)

Rat serum were stored at −80°C until analysis. ELISA was performed using the Interleukin Assay Kit as manufacturer’s instructions. Briefly, samples or standards were added to the corresponding wells, polystyrene 96-well plates were pre-coated for 30 min at 37°C and washed. Incubation with biotinylated detection antibody for 1h at RT. After, the 96-well plates were incubated with HRP-Avidin for 20 min at RT. The developer TMB solution were added and incubate for 30 minutes at room temperature in the dark. Microplate reader was used to detect the signals at 450 nm.

### Statistical analysis

SPSS 25.0 software (IBM, Armonk, NY, United States) was used for statistical analysis. Colon length, ATP content, immunohistochemistry, immunofluorescence, RT-qPCR, Western blot and ELISA analysis data were normally distributed; thus, they are presented as the mean ± standard deviation ($\bar{x}\pm s$) and were analyzed by one-way ANOVA with Bonferroni’s multiple comparison test. Colon length, ATP content, immunofluorescence, RT-qPCR (except that for NLRP3, caspase-1), Western blot analysis data (except that for IL-18) and ELISA (IL-18) were analyzed by the least significant difference (LSD) test. The Games-Howell test was used to analyze immunohistochemistry, RT-qPCR (NLRP3, caspase-1), Western blot analysis data (IL-18) and ELISA (IL-1β) data. CMDI and histopathological score data were not normally distributed; thus, they are expressed as the median (*P*_*25*_, *P*_*75*_) and were analyzed by the nonparametric Kruskal-Wallis *H* test. *P*_*adj*_ < 0.05 was considered statistically significant.

## Results

### HPM reduced the colon macroscopic damage index and alleviated colon shortening in CD rats

**Fig 3:**
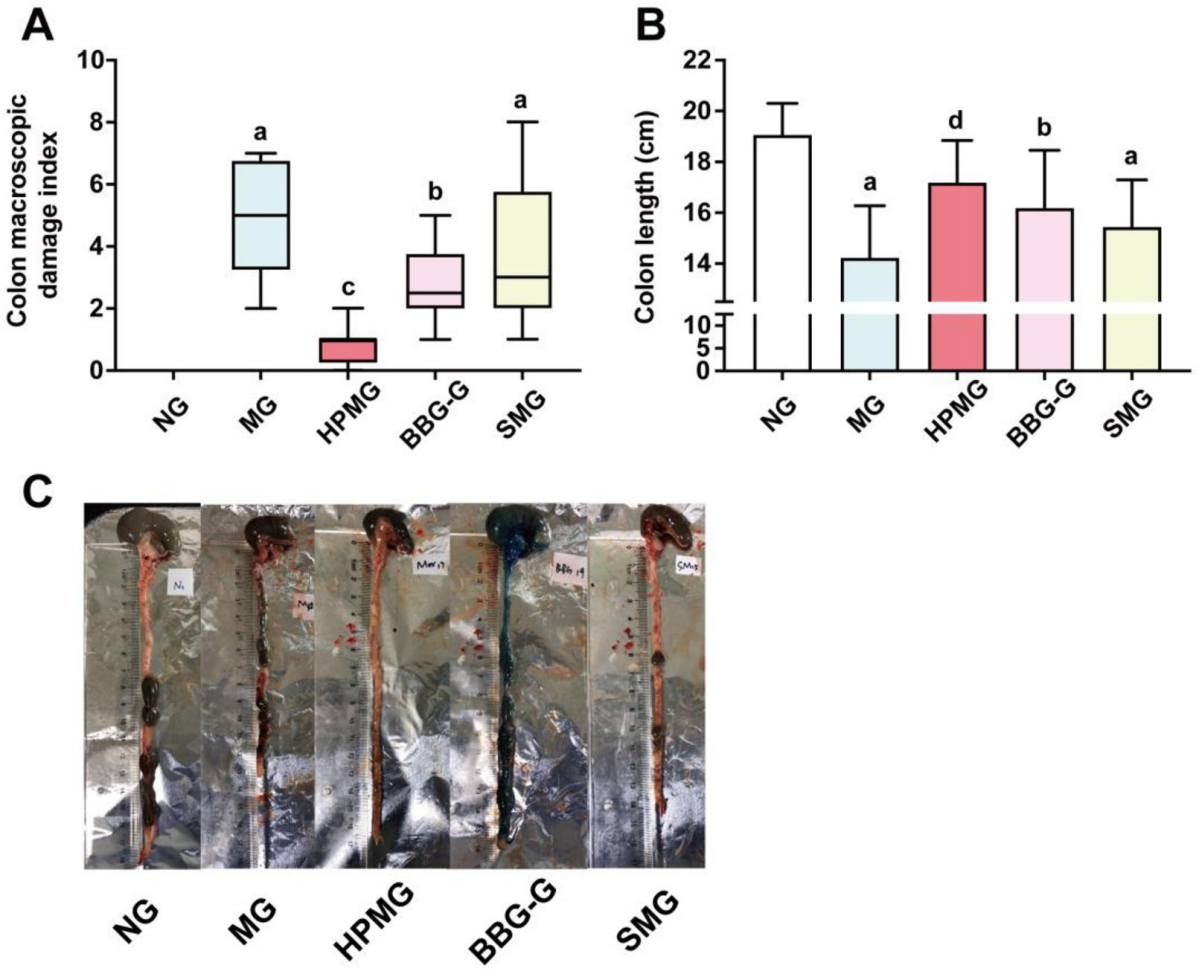
CMDIs and colon length in each group. **Note:** (A) CMDIs in each group. (B) Colon length in each group. (C) Colon images in each group. ^a^*P* < 0.01, ^b^*P* < 0.05 vs the NG; ^c^*P*<0.01, ^d^*P*<0.05 vs the MG. NG: normal group; MG: model group; HPMG: herb-partitioned moxibustion group; BBG-G: brilliant blue G group; SMG: sham moxibustion group. Data are presented as the Median (*P*_*25*_, *P*_*75*_) in (A) and ($\bar{x}\pm s$) in (B), n = 8.

Upon visual inspection, we found that the colons of rats in the NG were light pink, did not adhere to the surrounding tissues, and exhibited resilient intestinal walls. In contracts, the colons of rats in the MG, BBG-G and SMG exhibited different degrees of adhesion, dark red or brown and thickened intestinal walls, submucosal hyperemia, edema, and partially visible scattered ulcers; additionally, colon macroscopic damage indexes (CMDIs) were significantly increased in rats in the MG, BBG-G and SMG compared with those in the NG (all *P* < 0.05) (Fig 3A). The colons of rats in the HPMG exhibited mild adhesion to the surrounding tissues, slightly thickened intestinal walls, and ulcers that showed a tendency to recover, and rats in the HPMG had significantly lower CMDIs than those in the MG (*P* < 0.01) (Fig 3A). The CMDIs of the SMG and BBG-G were not significantly different (both *P* > 0.05 vs. MG) (Fig 3A). The results confirmed that HPM alleviated colon injury in CD rats. Compared with those of rats in the NG, the colons of rats in the MG, BBG-G and SMG were significantly shorter (all *P* < 0.05) (Fig 3B and 3C). Compared with those in rats in the MG, the colons of rats in the HPMG were significantly longer (*P* < 0.05). We found that colon shortening was improved by HPM treatment in CD rats.

### HPM reduced colon histopathological scores in CD rats

**Fig 4:**
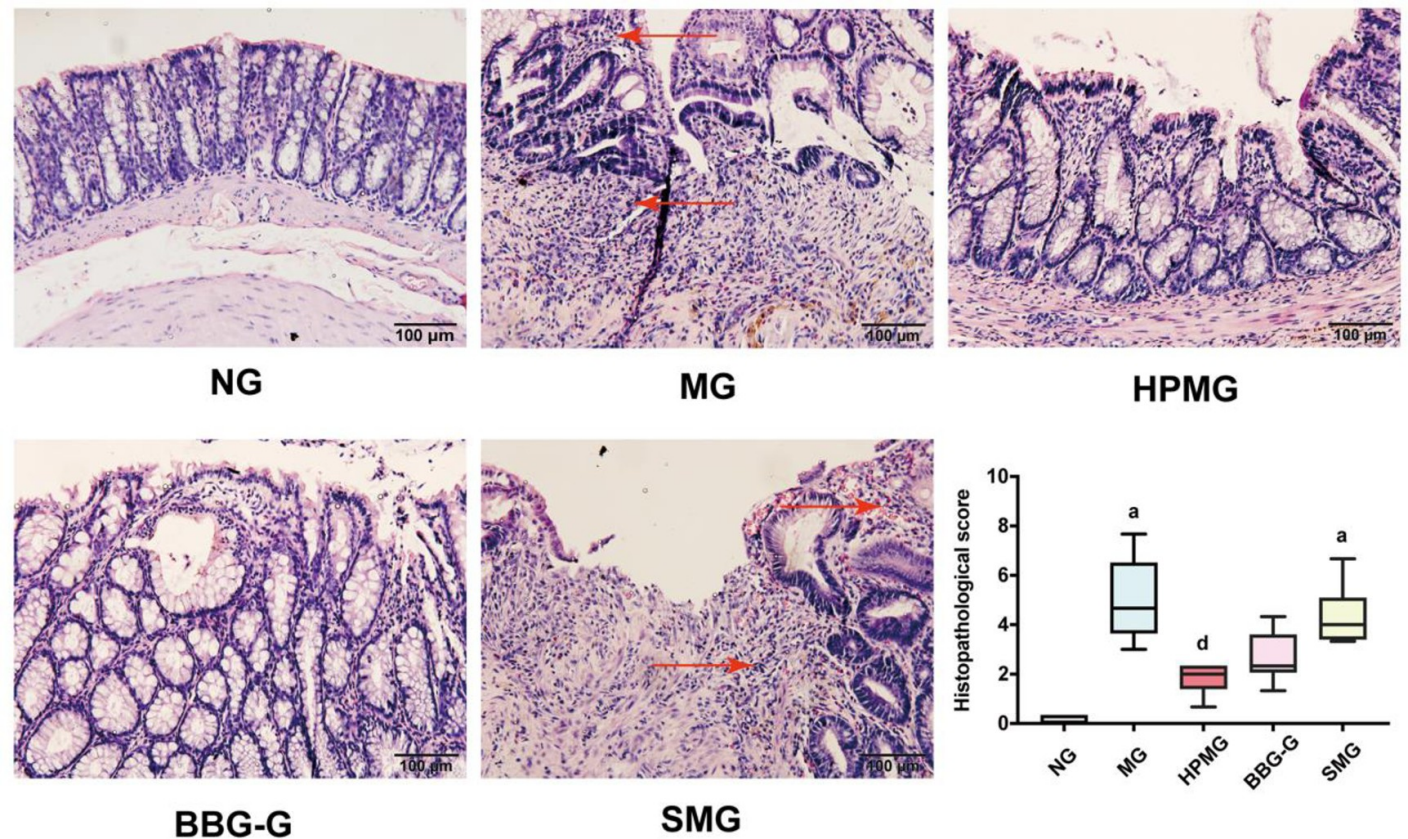
Morphological observation and histopathological scores of colon tissues from each group. **Note:** ^a^*P* < 0.01 vs the NG; ^d^*P*<0.05 vs the MG. NG: normal group; MG: model group; HPMG: herb-partitioned moxibustion group; BBG-G: brilliant blue G group; SMG: sham moxibustion group. The red arrow indicates inflammatory cell infiltration. Data are presented as the Median (*P*_*25*_, *P*_*75*_). Scale bar: 100 *μ*m, n = 8.

Observation under an optical microscope (Fig 4) revealed that the colons of rats in the NG exhibited complete colonic tissue structure with an intact epithelial layer, goblet cells with regular morphology and a neat arrangement, and no submucosal hyperemia or edema. Compared with those of the NG, the histopathological scores of the MG and the SMG were significantly higher (both *P* < 0.01). In the MG and SMG, colon injury was severe; in the colons, the epithelial layer was missing and local goblet cell destruction, mucosal damage, ulcer formation, a large number of inflammatory cells in the mucosa and submucosa, submucosal hyperemia and edema were observed. Colonic tissue injury and histopathological scores were significantly improved in the HPMG compared to the MG (*P* < 0.05). In the HPMG, epithelial tissues, goblet cells and glands were repaired, and mild or moderate infiltration of inflammatory cells into the lamina propria mucosa and submucosa and moderate edema in the submucosal connective tissues were observed. Although the damage to colon tissues was improved in the BBG-G compared to the MG, histopathological scores were not significantly different between these groups (*P* > 0.05), epithelial tissue damage was partially repaired, and the number of fibrotic ulcers was not changed. These results indicated that HPM treatment can reverse colon damage in CD rats.

### HPM decreased ATP content and the colonic protein and mRNA expression of colonic P2X7R in CD rats

**Fig 5:**
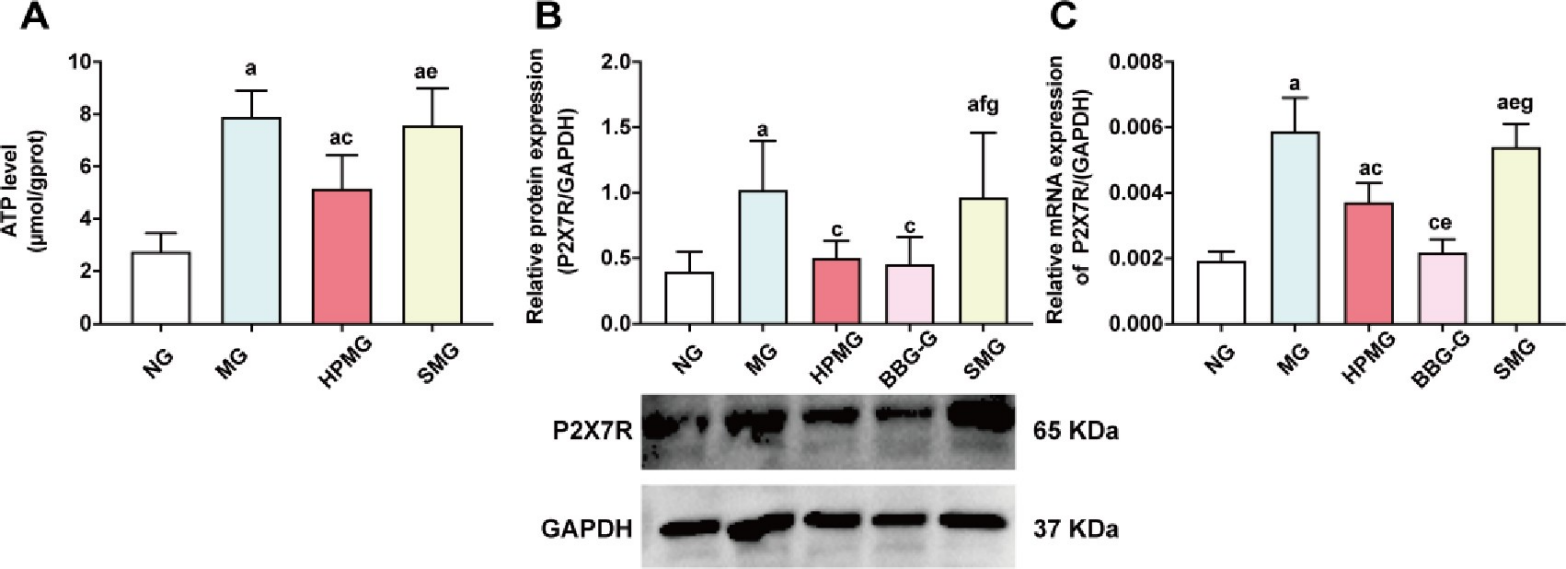
ATP content and the protein and mRNA expression of P2X7R protein in colon tissues from each group. **Note:** (A) The ATP content in colon tissues in each group. (B) Western blot analysis of P2X7R protein in colons in each group. (C) RT-qPCR analysis of P2X7R mRNA in colons in each group. ^a^*P* < 0.01 vs the NG; ^c^*P*<0.01 vs the MG; ^e^*P*<0.01,^f^*P*<0.05 vs the HPMG; ^g^*P*<0.01 vs the BBG-G. NG: normal group; MG: model group; HPMG: herb-partitioned moxibustion group; BBG-G: brilliant blue G group; SMG: sham moxibustion group. Data are presented as the ($\bar{x}\pm s$), n = 8.

Compared with that in the NG, colonic ATP content was significantly increased in the MG, HPMG and SMG (all *P* < 0.01) (Fig 5A). Compared with that in the MG, colonic ATP content was significantly decreased in the HPMG (*P* < 0.01) (Fig 5A). Compared with that in the HPMG, colonic ATP content was significantly higher in the SMG (*P* < 0.01) (Fig 5A). The data suggested that HPM decreased colonic ATP content in CD rats.

Western blot analysis (Fig 5B) showed that compared with that in the NG, the colonic expression of P2X7R in was significantly increased in the MG and SMG (both *P* < 0.01). Compared with that in the MG, colonic P2X7R expression was significantly reduced in the HPMG and BBG-G (both *P* < 0.01). Compared with that in the HPMG and BBG-G, colonic P2X7R expression was significantly higher in the SMG (both *P* < 0.05). There was no significant difference in P2X7R expression between the BBG-G and HPMG (*P* > 0.05). The results showed that both HPM and BBG inhibited colonic P2X7R expression in CD rats.

Moreover, RT-qPCR analysis (Fig 5C) showed that compared with that in the NG, the mRNA level of P2X7R in colon tissues was increased in the MG, HPMG and SMG (all *P* < 0.01). Compared with that the MG, the mRNA level of P2X7R in colon tissues was significantly reduced in the HPMG and BBG-G (both *P* < 0.01). The P2X7R mRNA level in colon tissues was significantly lower in the BBG-G than in the HPMG (*P* < 0.01), and the P2X7R mRNA level was significantly higher in the SMG than in the HPMG (*P* < 0.01). Compared with that in the BBG-G, the P2X7R mRNA level in colon tissues was significantly increased in the SMG (*P* < 0.01). The results indicated that both HPM and BBG reduced the increase in colonic P2X7R mRNA expression in CD rats.

### HPM inhibited the colonic protein and mRNA expression of Pannexin-1 in CD rats

**Fig 6:**
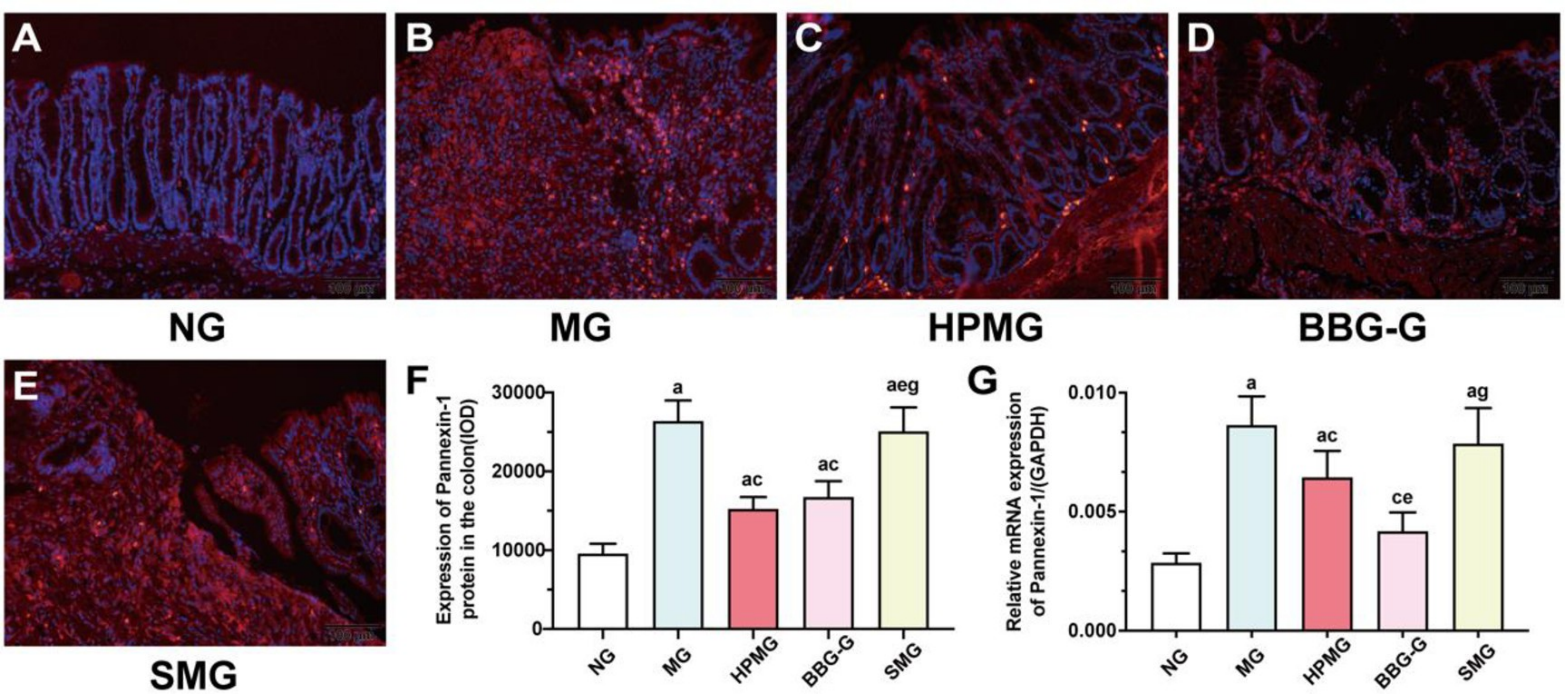
The protein and mRNA expression of Pannexin-1 in colon tissues from each group. **Note:** (A~E) Immunofluorescence of Pannexin-1 in colon tissues in each group with Pannexin-1 (red) and nucleus (blue). (F) IOD analysis of Pannexin-1 in colon tissues in each group. (G) RT-qPCR analysis of Pannesin-1 mRNA in colon tissues in each group. ^a^*P* < 0.01 vs the NG; ^c^*P*<0.01 vs the MG; ^e^*P*<0.01,^f^*P*<0.05 vs the HPMG; ^g^*P*<0.01 vs the BBG-G. NG: normal group; MG: model group; HPMG: herb-partitioned moxibustion group; BBG-G: brilliant blue G group; SMG: sham moxibustion group. Data are presented as the ($\bar{x}\pm s$). Scale bar: 100 *μ*m, n = 8.

The immunofluorescence analysis revealed that the NG had sparse Pannexin-1 expression in colons (Fig 6A), while the MG and SMG had a great many Pannexin-1 expression in colons (Fig 6B–6E). The HPMG and BBG-G had mild Pannesin-1 expression in colons (Fig 6C and 6D). Compared with the NG, the expression of Pannexin-1 in colons was significantly increased in the MG, HPMG, BBG-G and SMG (all P < 0.01) (Fig 6F). Compared with that in the NG, the colonic expression of Pannexin-1 was significantly increased in the MG, HPMG, BBG-G and SMG (all *P* < 0.01) (Fig 6F). Compared with that in the MG, the colonic expression of Pannexin-1 in the HPMG and BBG-G was significantly downregulated (both *P* < 0.01) (Fig 6F). The expression of Pannexin-1 in the colon was significantly higher in the SMG than in the HPMG and BBG-G (both *P* < 0.01) (Fig 6F). The results revealed that both HPM and BBG treatment downregulated the colonic expression of Pannexin-1 protein in CD rats.

Moreover, RT-qPCR analysis (Fig 6G) showed that compared with that in the NG, the Pannexin-1 mRNA level in colon tissues was significantly increased in the MG, HPMG and SMG (all *P* < 0.01). Compared with that in the MG, the mRNA level of Pannexin-1 in colon tissues was significantly reduced in the HPMG and BBG-G (both *P* < 0.01), but no significant difference was found between the MG and the SMG (*P* > 0.05). Compared with that in the HPMG, the Pannexin-1 mRNA level in colon tissues was significantly decreased in the BBG-G (*P* < 0.01), and the Pannexin-1 mRNA level was significantly higher in the SMG than in the BBG-G (*P*_BBG-G_ < 0.01). The results indicated that both HPM and BBG treatment inhibited the colonic mRNA expression of Pannexin-1 in CD rats and that the effect of BBG was more robust than that of HPM. Together, HPM may inhibit the abnormal activation of the NLRP3 inflammasome by regulating the P2X7R-Pannexin-1 signaling pathway.

### HPM downregulated the colonic protein and mRNA expression of NF-κBp65 in CD rats

**Fig 7:**
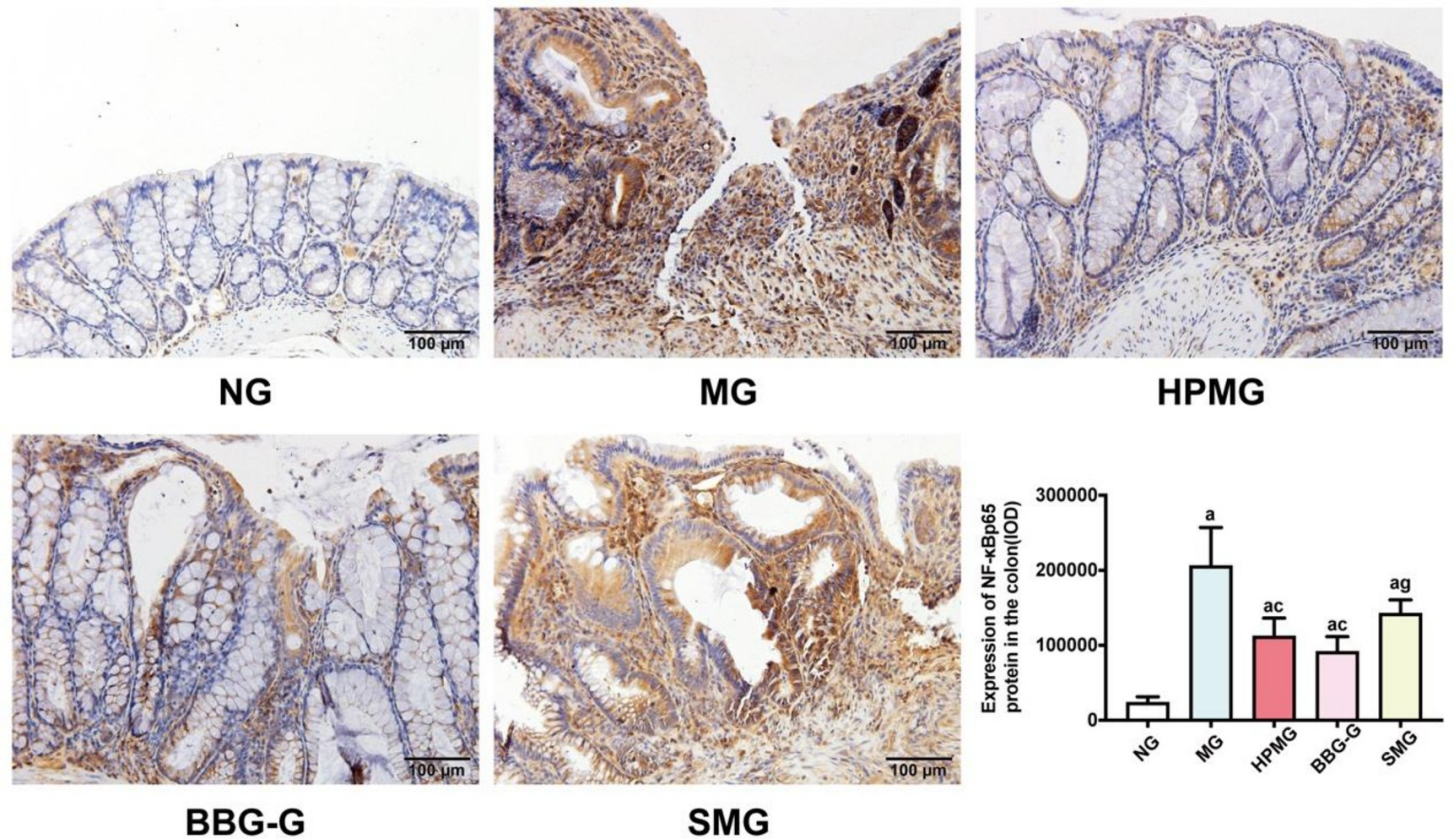
Images of NF-κBp65 (brown) immunostaining and corresponding IOD analysis of NF-κBp65 expression in colon tissues from each group. **Note:** ^a^*P* < 0.01 vs the NG; ^c^*P*<0.01 vs the MG; ^g^*P*<0.01 vs the BBG-G. NG: normal group; MG: model group; HPMG: herb-partitioned moxibustion group; BBG-G: brilliant blue G group; SMG: sham moxibustion group. Data are presented as the ($\bar{x}\pm s$). Scale bar: 100 *μ*m, n = 8.

We explored the effect of HPM on NF-κBp65 expression in CD rats. Immunohistochemical analysis (Fig 7) showed that there was more positive NF-κBp65 staining detected in the colonic mucosa and submucosa in the MG than in the NG, while both HPM and BBG reduced positive NF-κBp65 staining in the colonic mucosa and submucosa. IOD analysis of NF-κBp65 showed that compared with that in the NG, colonic NF-κBp65 expression in was significantly higher in the MG, HPMG, BBG-G and SMG (all *P* < 0.01). Compared with that in the MG, colonic NF-κBp65 expression was significantly reduced in the HPMG and BBG-G (both *P* < 0.01). However, NF-κBp65 expression was markedly increased in the SMG compared with the BBG-G (*P* < 0.01). The results suggested that both HPM and BBG treatment downregulated NF-κBp65 expression in the colons of CD rats.

**Fig 8:**
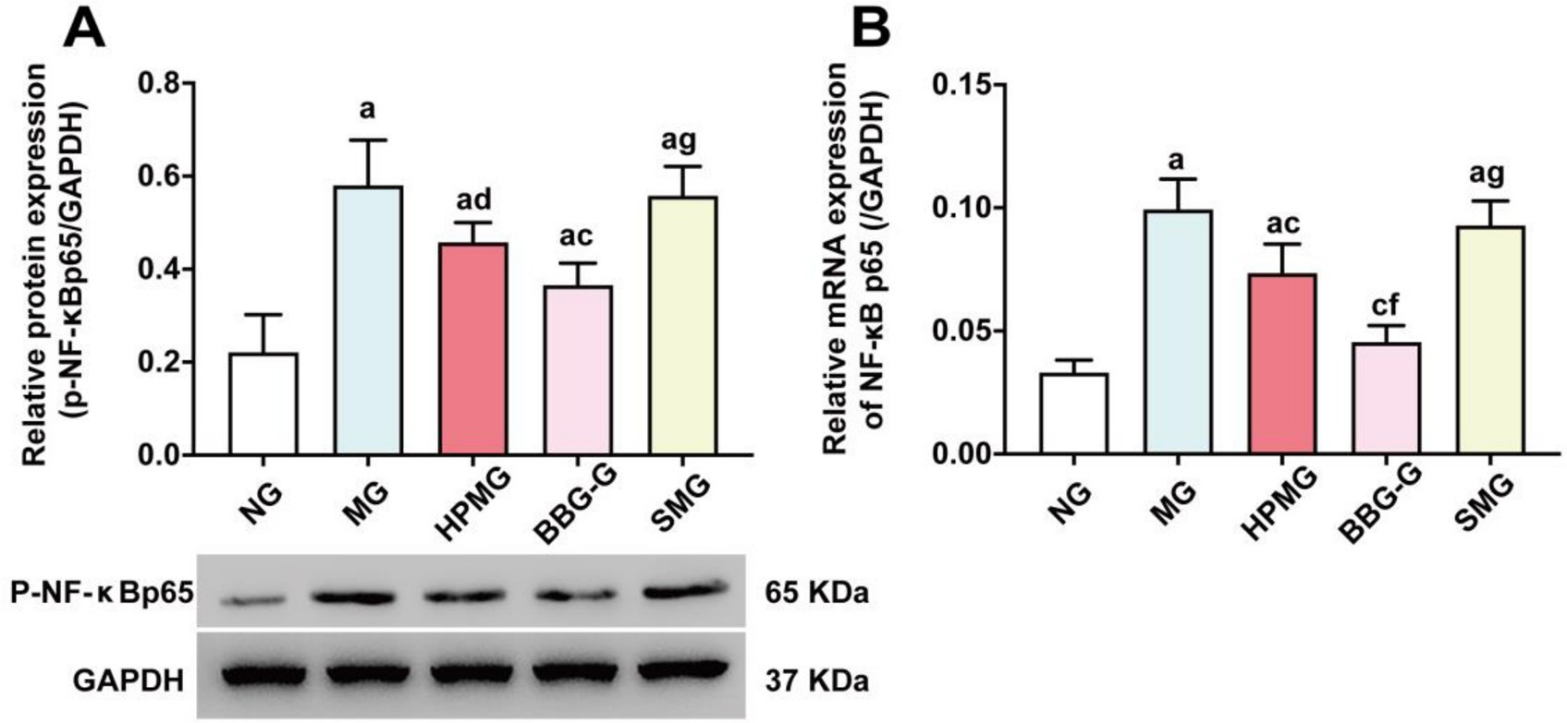
Protein expression of p-NF-κBp65 and mRNA expression of NF-κBp65 in colon tissues from each group. **Note:** (A) Western blot analysis of p-NF-κBp65 protein in colon homogenate in each group. (B) RT-qPCR analysis of NF-κBp65 mRNA in colon tissues in each group. ^a^*P* < 0.01, ^b^*P*<0.05 vs the NG; ^c^*P*<0.01 vs the MG; ^e^*P*<0.01,^f^*P*<0.05 vs the HPMG; ^g^*P*<0.01 vs the BBG-G. NG: normal group; MG: model group; HPMG: herb-partitioned moxibustion group; BBG-G: brilliant blue G group; SMG: sham moxibustion group. Data are presented as the ($\bar{x}\pm s$), n = 8.

Moreover, Western blot analysis (Fig 8A) showed that compared with that in the NG, the protein expression of p-NF-κBp65 in colon homogenates was significantly increased in the MG, HPMG, BBG-G and SMG (all *P* < 0.01). Compared with that in the MG, the protein expression of p-NF-κBp65 in colon homogenates was significantly reduced in the HPMG and BBG-G (both *P* < 0.05), but no significant difference was noted between the MG and the SMG (*P* > 0.05). The p-NF-κBp65 protein expression was significantly higher in the SMG than in the BBG-G (*P* < 0.01). The results indicated that both HPM and BBG inhibited the phosphorylation of NF-κBp65 protein in the colon tissues of CD rats.

Further RT-qPCR analysis (Fig 8B) revealed that compared with that in the NG, the NF-κBp65 mRNA level in colon tissues was significantly higher in the MG, HPMG and SMG (*P*_MG_ < 0.01, *P*_HPMG_ < 0.01, *P*_SMG_ < 0.01). Compared with that in the MG, the NF-κBp65 mRNA level in colon tissues was significantly lower in the HPMG and BBG-G (both *P* < 0.01), while no significant difference in the NF-κBp65 mRNA level was detected between the SMG and MG (*P* > 0.05). The colonic mRNA level of NF-κBp65 in the BBG-G was markedly lower than that in the HPMG (*P* < 0.05). Compared with that in the BBG-G, the colonic mRNA level of NF-κBp65 was significantly increased in the SMG (*P* <0.01). The results revealed that both HPM and BBG downregulated the expression of NF-κBp65 mRNA and that BBG treatment was more effective than HPM.

### HPM suppressed the protein expression of NLRP3, ASC, and caspase-1 in colon tissues from CD rats

**Fig 9:**
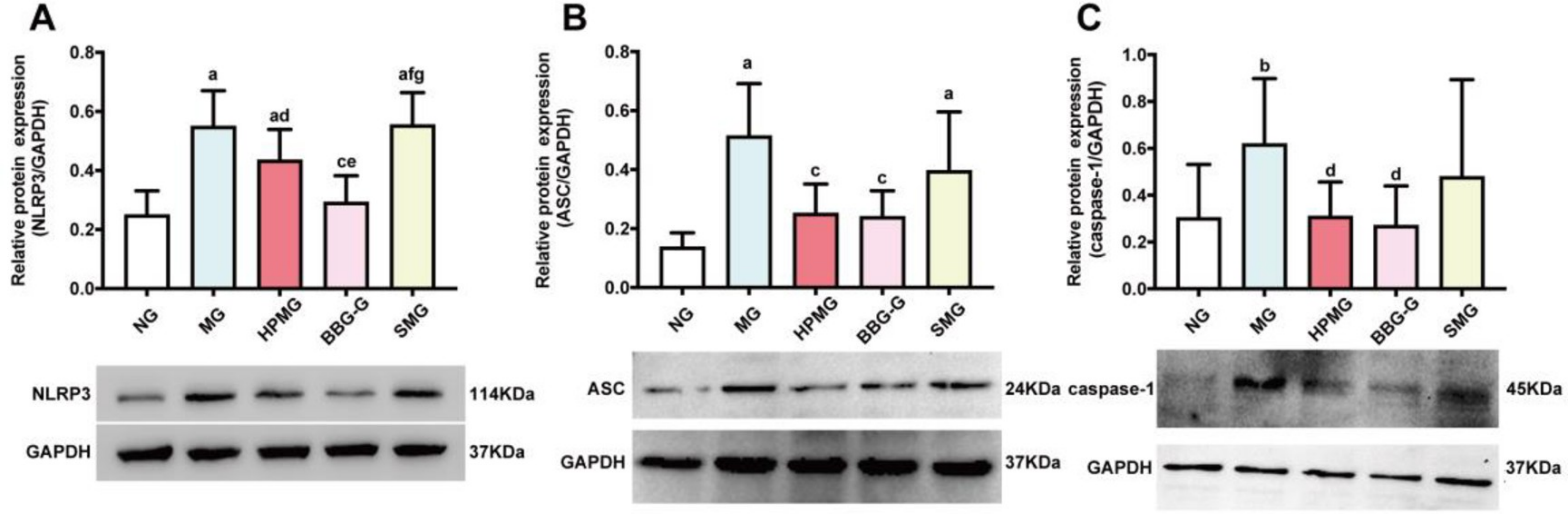
Protein expression of NLRP3, ASC, and caspase-1 in colon tissues from each group. **Note:** (A) Western blot analysis of NLRP3 protein in colon homogenate in each group. (B) Western blot analysis of ASC protein in colon homogenate in each group. (C) Western blot analysis of caspase-1 protein in colon homogenate in each group. ^a^*P* < 0.01, ^b^*P* <0.05 vs the NG; ^c^*P*<0.01, ^d^*P*<0.05 vs the MG; ^e^*P*<0.01,^f^*P*<0.05 vs the HPMG; ^g^*P*<0.01, ^h^*P*<0.05 vs the BBG-G. NG: normal group; MG: model group; HPMG: herb-partitioned moxibustion group; BBG-G: brilliant blue G group; SMG: sham moxibustion group. Data are presented as the ($\bar{x}\pm s$), n = 8.

Western blot analysis (Fig 9A) revealed that compared with that in the NG, the protein expression of NLRP3 in colon homogenates was significantly higher in the MG, HPMG, and SMG (all *P* < 0.01). Compared with that in the MG, NLRP3 protein expression in colon homogenates was significantly lower in the HPMG and BBG-G (*P*_HPMG_ < 0.05, *P*_BBG-G_ < 0.01). However, there was no significant difference in NLRP3 protein expression between the SMG and MG (*P* > 0.05). Compared with that in the HPMG, the protein expression of NLRP3 in colon homogenates was markedly downregulated in the BBG-G (*P* < 0.01), but was significantly upregulated in the SMG (*P* < 0.01). The protein expression of NLRP3 was also upregulated in the SMG compared with the BBG-G (*P* < 0.01). The results indicated that the protein expression of NLRP3 in the colon was inhibited by both HPM and BBG.

Moreover, analysis of ASC protein expression levels (Fig 9B) showed that, compared with that in the NG, the protein expression of ASC in colon homogenates was significantly increased in the MG and SMG (both *P* < 0.01). Compared with that in the MG, ASC protein expression in colon homogenates was significantly inhibited in the HPMG and BBG-G (both *P* < 0.01). No significant difference in ASC expression was observed between the MG and the SMG (*P* > 0.05). Our results suggested that both HPM and BBG downregulated the expression of ASC protein in the colon tissues of CD rats.

In addition, observation of the protein expression of caspase-1 (Fig 9C) showed that, compared with that in the NG, the protein expression of caspase-1 in colon homogenates was remarkably increased in the MG (*P* < 0.05). The expression of caspase-1 protein in colon homogenates was significantly decreased in the HPMG and BBG-G compared to the MG (both *P* < 0.05), but there was no significant difference in caspase-1 protein expression between the SMG and the MG (*P* > 0.05). The results revealed that both HPM and BBG inhibited the activation of the caspase-1 protein in the colons of CD rats.

### HPM suppressed the protein expression of IL-1β and IL-18 in the colon tissues of CD rats

**Fig 10:**
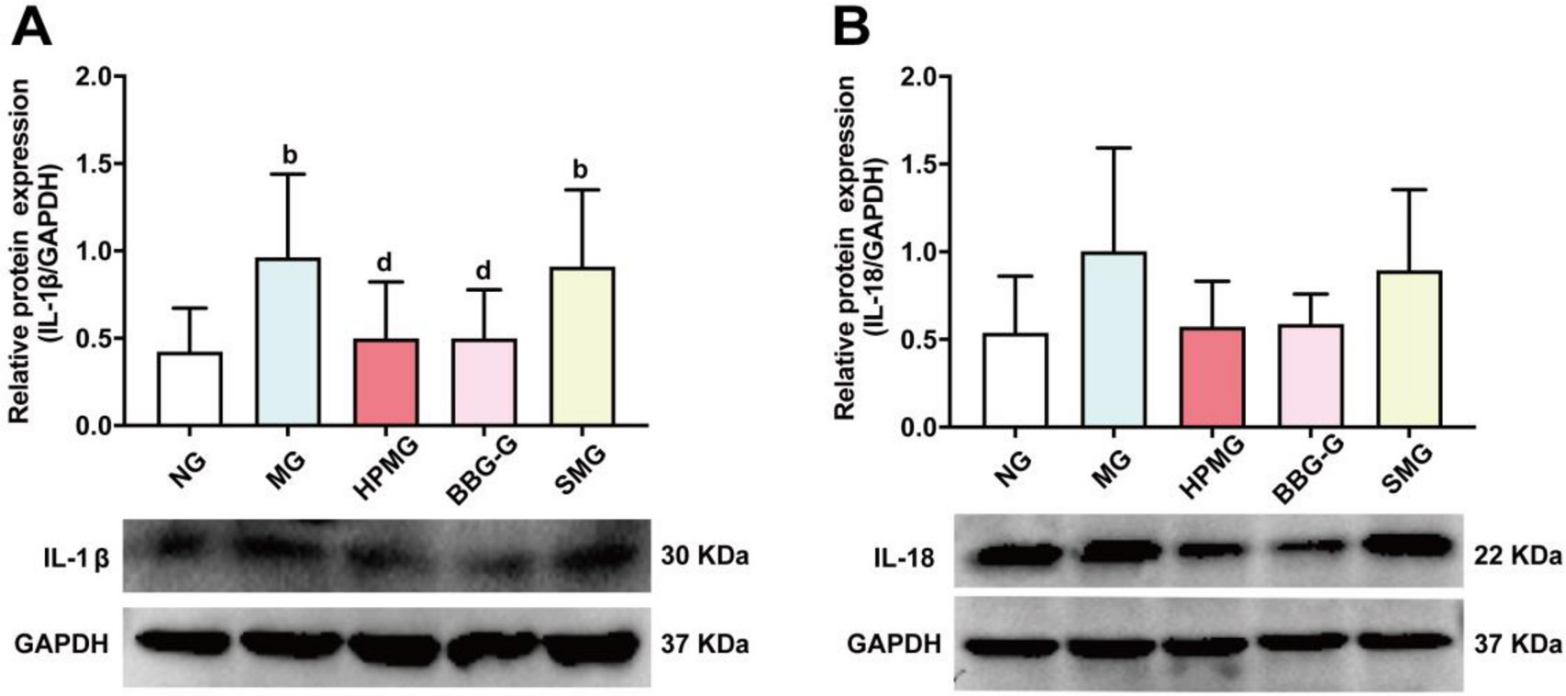
Protein expression of IL-1β and IL-18 in colon tissues from each group. **Note:** (A) Western blot analysis of IL-1β protein in colon homogenate in each group. (B) Western blot analysis of IL-18 protein in colon homogenate in each group. ^a^*P* < 0.01, ^b^*P* <0.05 vs the NG; ^d^*P*<0.05 vs the MG; ^f^*P*<0.05 vs the HPMG; ^h^*P*<0.05 vs the BBG-G. NG: normal group; MG: model group; HPMG: herb-partitioned moxibustion group; BBG-G: brilliant blue G group; SMG: sham moxibustion group. Data are presented as the ($\bar{x}\pm s$), n = 8.

We observed that the protein expression of IL-1β in colon homogenates was significantly higher in the MG and SMG than in the NG (Fig 10A; *P* < 0.05). However, compared with that in the MG, the protein expression of IL-1β in colon homogenates was considerably lower in the HPMG and BBG-G (both *P* < 0.05). The results demonstrated that both HPM and BBG reduced colonic IL-1β protein expression in CD rats.

We further evaluated IL-18 protein expression in the colon (Fig 10B). Compared with that in the NG, the protein expression of IL-18 protein was found to exhibit an increasing trend in colon homogenates from the MG and SMG, but the difference was not statistically significant (*P* > 0.05). The protein expression of IL-18 in colon homogenates was lower in the HPMG and BBG-G than in the MG, but the differences were not statistically significant (both *P* > 0.05). These results demonstrated that HPM and BBG had no significant effect on the regulation of IL-18 protein expression in the colons of CD rats.

## Discussion

Although the pathogenesis of CD remains unclear, susceptibility to CD is associated with abnormalities in NOD2 and the NLRP3 inflammasome [19]. At inflammatory sites, P2X7R can be activated by high concentrations of extracellular ATP. The combination of extracellular ATP and P2X7R allows the passage of cations, including Ca^2+^, Na^+^, and K^+^, across the plasma membrane [23, 24], recruits Pannexin-1 to mediate the opening of hemichannels [43], and then induces the activation of NF-κB, which causes NLRP3 transcription [27, 28, 44]. It has been reported that moxibustion intervention can have an anti-inflammatory effect by downregulating the protein expression of NF-κBp65 [15, 45]. In our previous study, we demonstrated that HPM can inhibit the abnormal activation of the NLRP3 inflammasome and the secretion of IL-1β in the colons of CD rats to promote the repair of inflammatory damage [29]. At the same time, we also detected the NLRP3 inflammasome, IL-1b and IL-18 by PCR and ELISA. the results indicated that HPM treatment inhibited the colonic mRNA expression of NLRP3, ASC and caspase-1, as well as the IL-1β and IL-18 contents in serum of CD rats (S1 and S2 Figs). However, the mechanism by which HPM regulates the NLRP3 inflammasome in CD rats is still unknown. Therefore, we established a CD rat model and explored whether HPM inhibits the excessive activation of NLRP3 inflammasomes through regulating the P2X7R-Pannexin-1 signaling pathway.

Previous studies showed that HPM treating (at Qihai (CV 6) and Tianshu (ST 25)) alleviated increased cell apoptosis in the intestinal epithelial barrier and improved intestinal epithelial tight junction integrity in CD [14, 15]. At the same time, another study reports that HPM treating (at Qihai (CV 6) and Tianshu (ST 25)) protected the mucosa and promoted repair of injured mucosa in ulcerative colitis [46]. Thus, in this study, an experimental model of CD was induced and the model animals were treated with HPM (at Qihai (CV 6) and bilateral Tianshu (ST 25)). A previous study discovered BBG treatment reduced P2X7R expression and suppressed NLRP3/IL-1β pathway signaling [47]. We therefore used P2X7R antagonist BBG as the positive control in our study. Our results revealed that compared with those of rats in the NG, the CMDIs and histopathological scores of CD rats were significantly increased (*P* < 0.01 vs. the NG) and the colon lengths of CD rats were significantly reduced (*P* < 0.01 vs. the NG). However, these defects were reversed by HPM treatment. Thus, our results showed that HPM can improve intestinal inflammatory damage in CD rats. It has been shown that a high concentration of extracellular ATP is closely associated with IBD pathogenesis [48], and we found that the ATP content in colon tissues was considerably increased in the MG compared with the NG group (*P* < 0.01). Conversely, HPM treatment significantly decreased ATP content in the colon tissues (*P* < 0.01 vs. the MG). Some studies have shown that inflammatory sites contain high extracellular ATP concentrations in vivo, and an elevated level of ATP release from the colon tissue was also noted in CD mice [49–52]. These indicate that ATP accumulates at the sites of injury and inflammation. Although we failed to detect the level of extracellular ATP, based on the above studies, we speculate that HPM may reduce the level of ATP release from the colon tissue, resulting in a decrease in the level of total ATP in colon homogenates in CD rats.

Extracellular ATP can promote the maturation of dendritic cells (DCs) and the release of inflammatory cytokines through the activation of the P2X7R-NF-κBp65 pathway [53], and the activation of P2X7R is associated with IBD pathogenesis [44]. In the present study, we found that the colonic protein expression levels of P2X7R, NF-κBp65 and p-NF-κBp65 and the colonic mRNA levels of P2X7R and NF-κBp65 were significantly increased in CD rats compared with rats in the NG (all *P* < 0.01). Furthermore, immunohistochemistry demonstrated strong positive NF-κBp65 staining in the colonic mucosa and submucosa in TNBS-induced CD rats. The above results indicate that P2X7R is activated by high concentrations of ATP in colon tissues, thereby promoting an increase in downstream NF-κBp65 expression in CD rats. In addition, the increase of ATP levels may be correlated with the increase of metabolism requirement during inflammation [54]. After BBG treatment, the protein expression of P2X7R, NF-κBp65 and p-NF-κBp65 and the mRNA levels of P2X7R and NF-κBp65 were significantly decreased in CD rats, which is consistent with previous reports [44]. We found that HPM and BBG exerted similar effects in regulating P2X7R and NF-κBp65. HPM also inhibited the protein expression of P2X7R, NF-κBp65 and p-NF-κBp65 and the mRNA levels of P2X7R and NF-κBp65 in the colons of CD rats. Previous studies showed that reducing the expression of P2X7R was efficacious in blocking IL-1β-mediated inflammation [55]. Therefore, we speculated that HPM may block P2X7R activation, thereby reducing the occurrence of NF-κB inflammatory cascade.

It has been reported that a high concentration of ATP can induce an increase in P2X7R expression, while P2X7R drives Pannexin-1 channel activation and then mediates the release of IL-1β [56]. This finding is consistent with our results. In CD rats, in addition to colonic ATP content and P2X7R expression being increased, the protein expression of Pannexin-1 and IL-1β and the mRNA level of Pannexin-1 were also significantly upregulated, which verified that P2X7R can activate Pannexin-1 channels to cause inflammation. In addition, Diezmos found that inhibiting the activation of P2X7R and Pannexin-1 suppresses the inflammatory cytokine-induced loss of tight junctions, crypt damage and increased cell permeability [57]. More importantly, we found that the increase in the expression of P2X7R and Pannexin-1 in the colons of CD rats was remarkably downregulated by HPM and BBG. Taken together, these results indicate that P2X7R and Pannexin-1 may be potential therapeutic targets for the treatment of IBD [57, 58] and that HPM can inhibit the activation of the P2X7R-Pannexin-1 signaling pathway, the upstream pathway of the NLRP3 inflammasome.

Studies have shown that susceptibility to CD is associated with abnormal activation of the NLRP3 inflammasome [19]. To further explore whether HPM regulates the NLRP3 inflammasome and its downstream inflammatory cytokines by affecting the P2X7R-Pannexin-1 pathway in CD rats, we examined the expression of proteins related to the NLRP3 inflammasome (NLRP3, ASC, and caspase-1) and IL-1β and IL-18, which are downstream of the NLRP3 inflammasome. We found that the protein expression levels of NLRP3, ASC, caspase-1, and IL-1β were considerably higher in the colons of CD rats than in those of normal group rats. The results further indicated that the activation of the NLRP3 inflammasome is associated with the pathogenesis of CD and that the NLRP3 inflammasome induces the production and secretion of IL-1β, resulting in the aggravation of colonic damage, which is consistent with previous reports [59, 60]. The activation of the NLRP3 inflammasome induces the secretion of IL-18 [20]. Although the protein expression of IL-18 was increased in the colons of CD rats compared with those of rats in the NG, the difference was not statistically significant (*P* > 0.05); the specific reasons for this lack of a significant difference require further study. After HPM or BBG treatment, the protein expression levels of NLRP3, ASC, caspase-1 and IL-1β were significantly decreased in the colons of CD rats. Thus, our findings indicate that both HPM and BBG can inhibit the excessive activation of the NLRP3 inflammasome and the production of the proinflammatory cytokine IL-1β. In addition, regulation of the NLRP3 inflammasome and IL-1β has been reported to be key for the treatment of IBD [20, 61]. Some studies have reported that ATP is shown to activate the P2X7R in dendritic cells and macrophages, leading to NLRP3 inflammasome activation [62, 63]. Therefore, we speculate HPM may modulate the NLRP3 inflammasome in dendritic cells or macrophages. In addition, since NLRP3 inflammasomes are also widely present in antigen-presenting cells, CD4⁺ T cells and B cells [64–66]. More in-depth exploration is required to reveal in which cells that HPM regulates the NLRP3 inflammasome.

In summary, this study shows that HPM, a traditional Chinese therapy, can block the abnormal activation of the NLRP3 inflammasome by inhibiting the P2X7R-Pannexin-1 signaling pathway, thereby reducing the release of the downstream inflammatory cytokine IL-1β and ultimately suppressing colonic inflammation in CD rats. However, there are some limitations to this study. Activation of the NLRP3 inflammasome did not significantly increase the expression of IL-18 in CD rats, and the specific reasons for this lack of a significant difference require further study. Since there is no validation cohort in this study, further studies are needed to investigate the regulatory effect of the P2X7R-Pannexin-1 pathway signaling pathway on the NLRP3 inflammasome when CD rats overexpress P2X7R in the future. We showed that HPM suppresses NLRP3 inflammasome activation via the regulation of the P2X7R-Pannexin-1 pathway, but whether HPM can affect the NLRP3 inflammasome in CD rats through other pathways or negative regulatory mechanisms requires in-depth exploration. In addition, this study only investigated the regulatory effect of HPM on the P2X7R-Pannexin-1 signaling pathway and NLRP3 inflammasome in a CD rat model. Further clinical studies are required to verify our findings in the future.

## Supporting information

S1 Table CMDI scoring. (DOCX)

S2 Table Histopathological scoring. (DOCX)

S1 Fig The mRNA expression of NLRP3, ASC and caspase-1 in colon tissues. (DOCX)

S2 Fig The secretion of IL-1β and IL-18 in serum. (DOCX)

S1 Raw images (TIF)
