# Supplementary material for: Herb-partitioned moxibustion alleviates colonic inflammation in Crohn’s disease rats by inhibiting hyperactivation of the NLRP3 inflammasome via regulation of the P2X7R-Pannexin-1 signaling pathway
Source: PLoS One. 2021 May 27;16(5):e0252334. doi: 10.1371/journal.pone.0252334 (PMC8158928; doi:10.1371/journal.pone.0252334)
Supplement: S1 Table — (DOCX) [file pone.0252334.s001.docx]

*S1 Table CMDI Scoring*

| Colon macroscopic damage index | | Score | |
| --- | --- | --- | --- |
| Colon adhesion | No adhesion | 0 | |
|  | Mild adhesion | 1 | |
|  | Severe adhesion | 2 | |
| Ulcer and inflammation | No ulcer and inflammation | 0 | |
|  | Local congestion without ulcer | 1 | |
|  | 1 ulcer without congestion or bowel wall thickening | 2 | |
|  |  |  | |
|  | 1 ulcer with inflammation | 3 | |
|  | 2 ulcers and inflammation | 4 | |
|  | > 2 ulcers with inflammation or inflammation area > 1 cm | | 5 |
|  | Ulcer and/or inflammation area = 2cm, One more damage, plus 1 | 6~8 | |
|  |  |  | |
|  |  |  | |
