# Supplementary material for: Herb-partitioned moxibustion alleviates colonic inflammation in Crohn’s disease rats by inhibiting hyperactivation of the NLRP3 inflammasome via regulation of the P2X7R-Pannexin-1 signaling pathway
Source: PLoS One. 2021 May 27;16(5):e0252334. doi: 10.1371/journal.pone.0252334 (PMC8158928; doi:10.1371/journal.pone.0252334)
Supplement: S2 Table — (DOCX) [file pone.0252334.s002.docx]

*S2 Table* *Histopathological Scoring*

| Histopathological manifestation | | Score |
| --- | --- | --- |
| Ulcer | No ulcer | 0 |
|  | Ulcer area<3 mm | 1 |
|  | Ulcer area>3 mm | 2 |
| Inflammation | No inflammation | 0 |
|  | Mild inflammation | 1 |
|  | Moderate inflammation | 2 |
|  | Severe inflammation | 3 |
| Lesion depth | No lesion | 0 |
|  | Submucosa | 1 |
|  | Muscularis propria | 2 |
|  | Serosal layer | 3 |
| Fibrosis | No fibrosis | 0 |
|  | Mild fibrosis | 1 |
|  | Severe fibrosis | 2 |
|  |  |  |
