# Supplementary material for: Herb-partitioned moxibustion alleviates colonic inflammation in Crohn’s disease rats by inhibiting hyperactivation of the NLRP3 inflammasome via regulation of the P2X7R-Pannexin-1 signaling pathway
Source: PLoS One. 2021 May 27;16(5):e0252334. doi: 10.1371/journal.pone.0252334 (PMC8158928; doi:10.1371/journal.pone.0252334)
Supplement: S1 Fig — (DOCX) [file pone.0252334.s003.docx]

*S1 Fig* *The mRNA expression of NLRP3, ASC and caspase-1 in colon tissues*

**
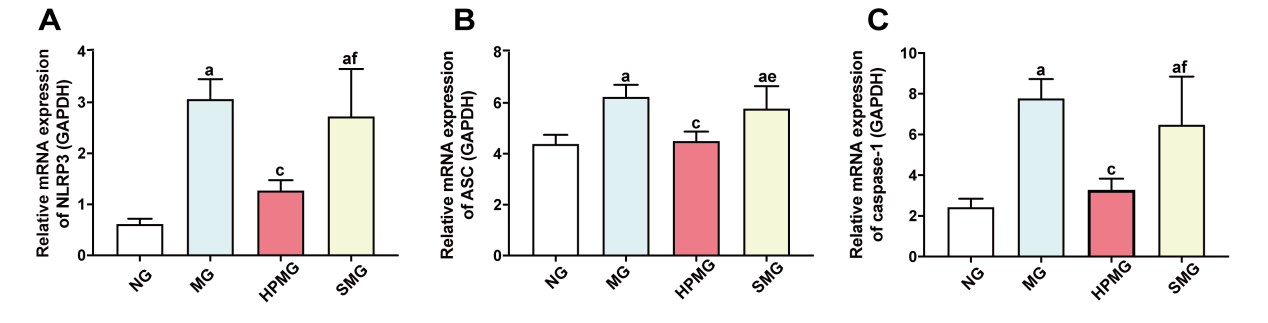
**

**S1 Fig** (A) RT-qPCR analysis of NLRP3 mRNA. (B) RT-qPCR analysis of ASC mRNA. (C) RT-qPCR analysis of caspase-1 mRNA. ^a^*P* < 0.01, ^b^*P* < 0.05 vs the NG; ^c^*P*<0.01 vs the MG; ^e^*P*<0.01, ^f^*P*<0.05 vs the HPMG. NG: normal group; MG: model group; HPMG: herb-partitioned moxibustion group; SMG: sham moxibustion group. Data are presented as the (). n = 8.
