# Supplementary material for: Herb-partitioned moxibustion alleviates colonic inflammation in Crohn’s disease rats by inhibiting hyperactivation of the NLRP3 inflammasome via regulation of the P2X7R-Pannexin-1 signaling pathway
Source: PLoS One. 2021 May 27;16(5):e0252334. doi: 10.1371/journal.pone.0252334 (PMC8158928; doi:10.1371/journal.pone.0252334)
Supplement: S2 Fig — (DOCX) [file pone.0252334.s004.docx]

*
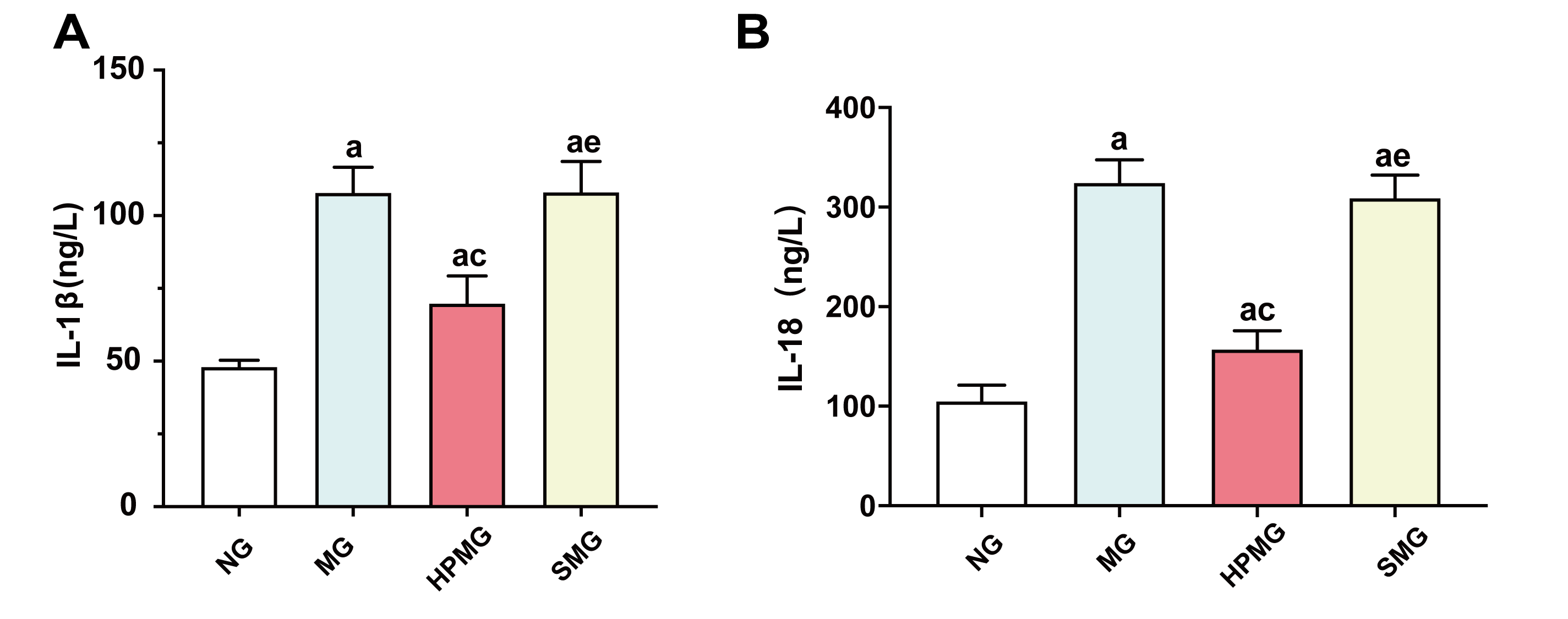
S2 Fig The secretion of IL-1β and IL-18 in serum*

**Fig R2** (A) ELISA analysis of IL-1β content. (B) ELISA analysis of IL-18 content. ^a^*P* < 0.01vs the NG; ^c^*P*<0.01 vs the MG; ^e^*P*<0.01 vs the HPMG. NG: normal group; MG: model group; HPMG: herb-partitioned moxibustion group; SMG: sham moxibustion group. Data are presented as the (). n = 8.
