# Supplementary figures and images for: Herb-partitioned moxibustion alleviates colonic inflammation in Crohn’s disease rats by inhibiting hyperactivation of the NLRP3 inflammasome via regulation of the P2X7R-Pannexin-1 signaling pathway
Source: PLoS One. 2021 May 27;16(5):e0252334. doi: 10.1371/journal.pone.0252334 (PMC8158928; doi:10.1371/journal.pone.0252334)

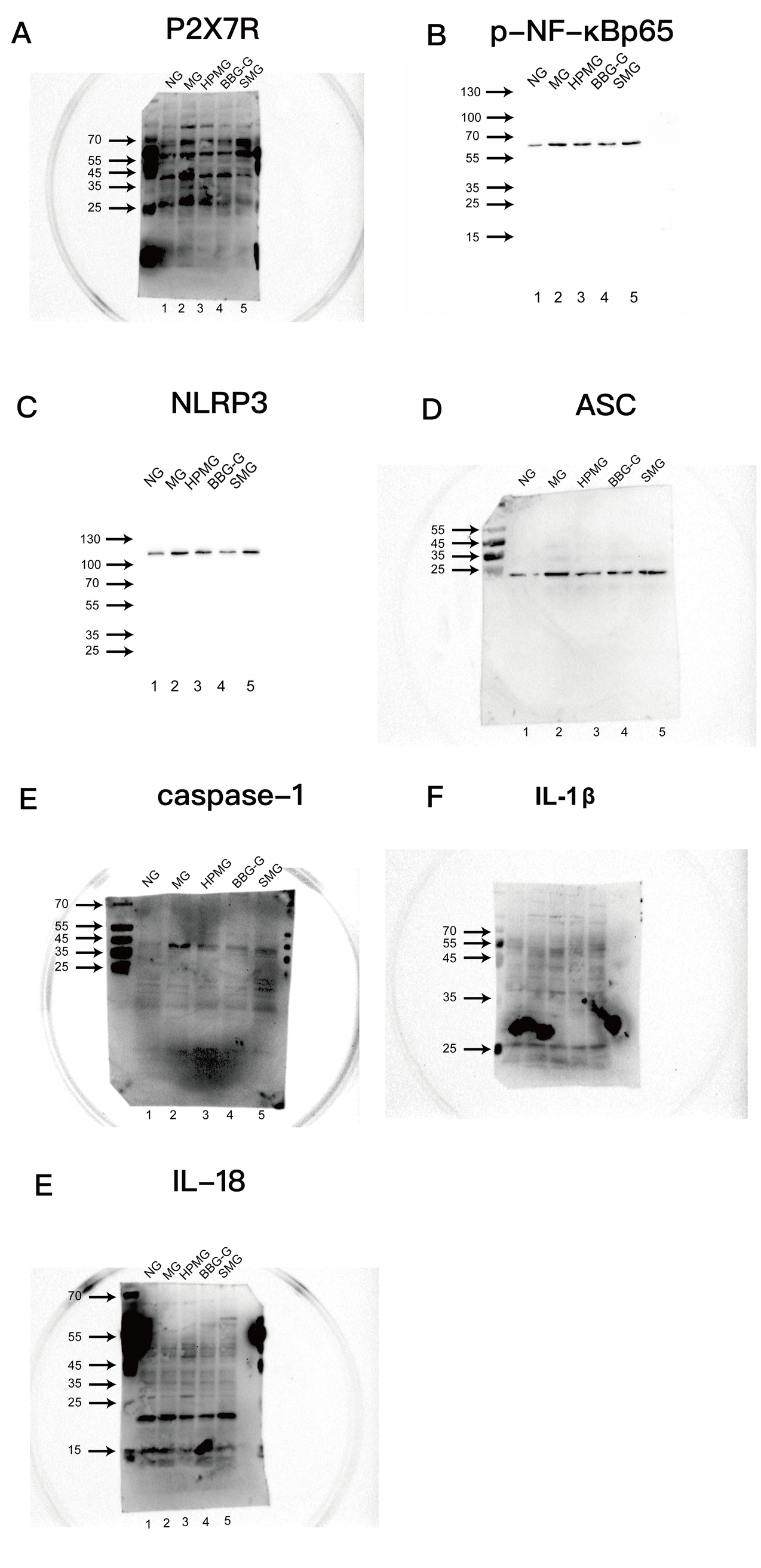

Supplement: S1 Raw images — (TIF) [file pone.0252334.s005.tif]
